# Supplementary material for: Salmonella Pathogenicity Island 1 (SPI-1): The Evolution and Stabilization of a Core Genomic Type Three Secretion System
Source: Microorganisms. 2020 Apr 16;8(4):576. doi: 10.3390/microorganisms8040576 (PMC7232297; doi:10.3390/microorganisms8040576)
Supplement: Supplementary file 1 [file microorganisms-08-00576-s001.pdf]

microorganisms

MDPI

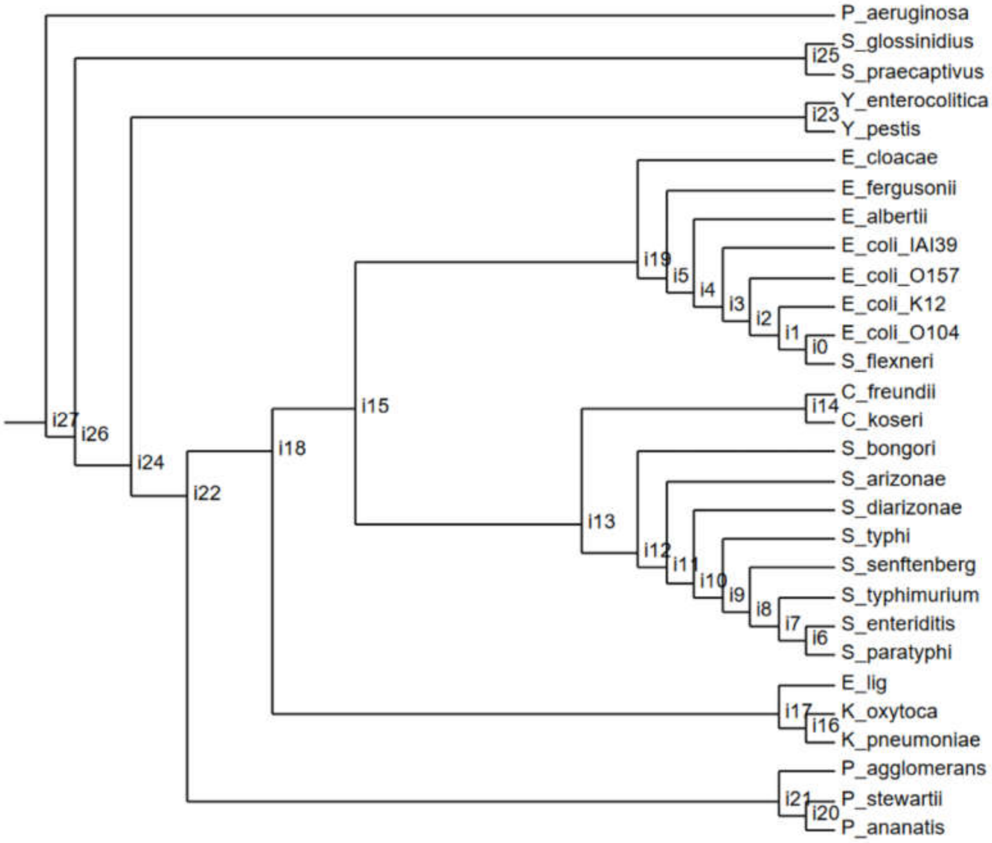

Supplementary Figure S1. Cladogram of bacterial strains used as input for xenoGI analysis. Phylogenetic relationships were determined by PATRIC [46]. Node labels refer to the hypothetical most recent common ancestor and are referenced in **Table S1.**

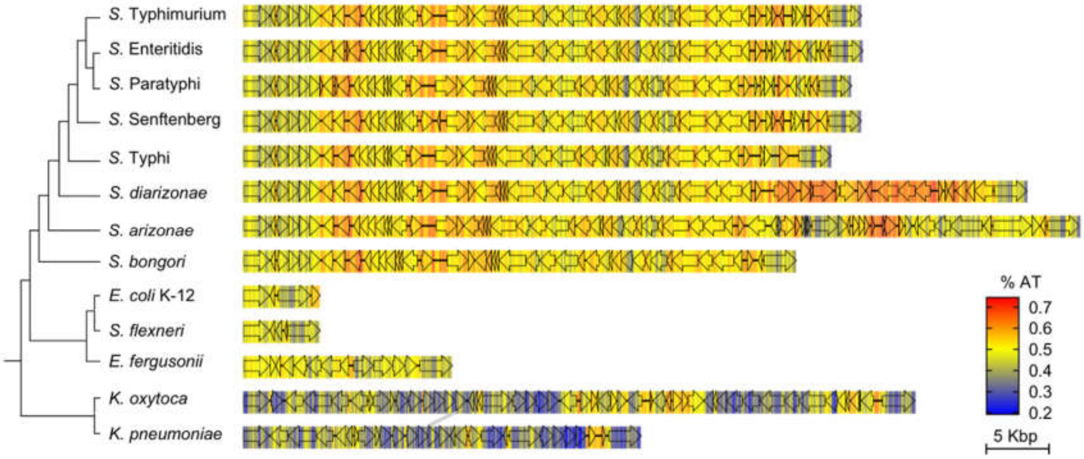

Supplementary Figure S2. AT content values of the *fhlA*/-/*mutS* locus overlaid as a heatmap on genomic islands in Enterobacteriaceae. Heatmap values were generated with a 100 base sliding window using Geneious R11 [52].

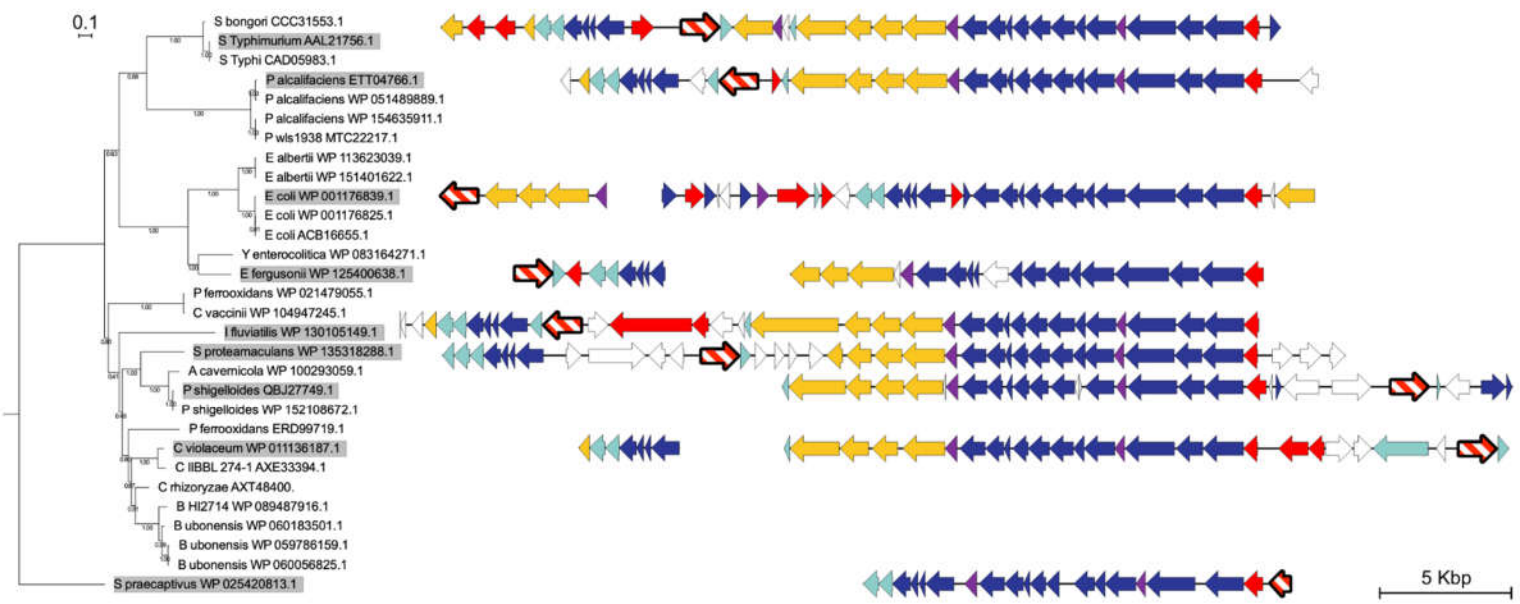

Supplementary Figure S3. HilA phylogeny alongside genomic context for *hilA* in various T3SSs. Best blastx hits covering at least 80% of the query for *S. enterica* Typhimurium LT2 *hilA* sequences were identified and one representative protein sequence was selected from each species to capture phylogenetic diversity. *S. bongori*, *S. enterica* Typhi and *S. enterica* Typhimurium HilA sequences were included as representatives from the *Salmonella* clade Protein sequences were aligned using MUSCLE and phylogeny was built using a maximum-likelihood model LG+G+I with 1000 bootstrap replicates. *hilA* genomic context was examined for select strains in grey boxes; *hilA* is illustrated in bold with white and red hashes and genes are coloured according to function (see **Figure 1** legend).

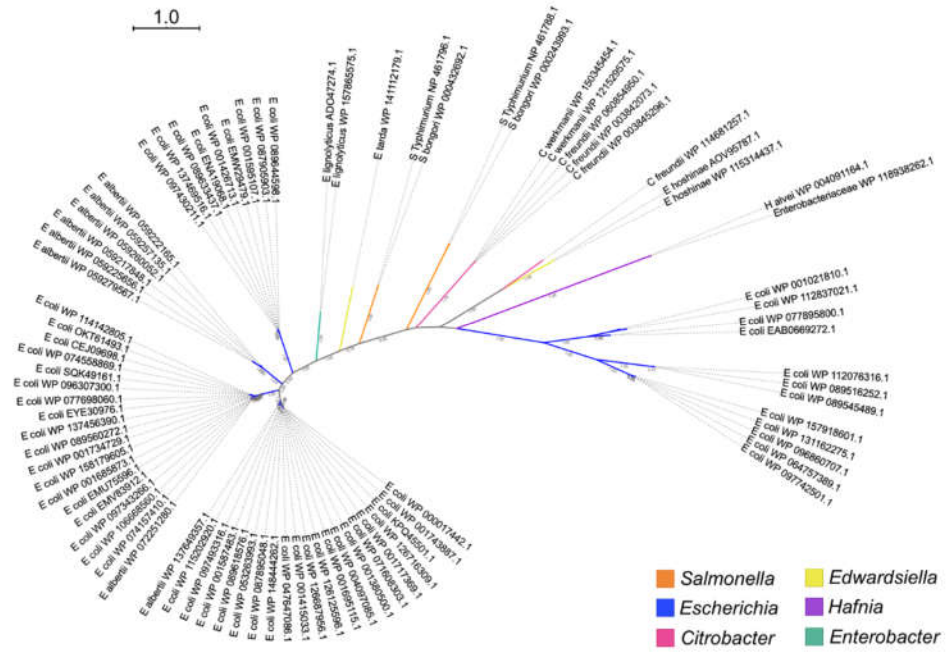

Supplementary Figure S4. Unrooted HilCD phylogeny of homologs found outside the *Salmonella* clade. Bootstrap support values over 0.70 are shown. Nodes are coloured by genus. *S. bongori* and *S. enterica* Typhimurium HilC and HilD sequences were included as representatives from the *Salmonella* clade. The top 100 best blastx hits covering at least 80% of the query for *S. enterica* Typhimurium LT2 HilC and HilD sequences were aligned using MUSCLE and phylogeny was built using a maximum-likelihood model JTT+G with 1000 bootstrap replicates. Both HilC and HilD searches returned 76 hits; of these, 72 proteins were present in both HilC and HilD top hit lists, with 4 unique proteins matching HilC and 4 unique proteins matching HilD.

Supplementary Table S1. Summary of xenoGI island classification for genes at the *fhlA*/-/*mutS* locus.

Organism	Has *ygbA*?	At *fhlA*/-/*mutS* locus?	Island ID[1]	Has *sitABCD*?	At *fhlA*/-/*mutS* locus?	Island ID[1]	Unique island or part of another?	Has *mutS*?	Island ID[1]	Has *pphB*?	Island ID[1]
C. freundii	y	y	21122	y	y	6307	Unique	y	812	n	-
C. koseri	y	y	21122	y	y	6307	Unique	y	812	n	-
E. albertii	y	y	21122	n	-	-	-	y	812	y	25098
E. coli IAI39	y	y	21122	y	n	6584, 7904, 5348	*sitCD* grouped with another, *sitA* grouped with another	y	812	y	25098
E. coli K12	y	y	21122	n	-	-	-	y	812	y	25098
E. coli O104:H4	y	y	21122	n	-	-	-	y	812	y	25098
E. coli O157:H7	y	y	21122	n	-	-	-	y	812	y	25098
E. fergusonii	y	y	21122	n	-	-	-	y	812	y	25098
S. flexneri	y	y	21122	y	n	6585, 7906, 6309	*sitCD* grouped with another, *sitA* grouped with another	y	812	n	-
E. cloacae	n	-	-	n	-			y	812	n	-
E. ligonolyticus	y	y	21122	y	n	6311	Unique	y	812	n	-
K. oxytoca	y	y	37682	y	y	6307	Unique	y	812	n	-
K. pneumoniae	y	y	37682	y	y	6307	Unique	y	812	n	-
P. aeruginosa	n	-	-	n	-	-	-	y	812	n	-
P. agglomerans	n	-	-	n	-	-	-	y	812	n	-
P. ananatis	n	-	-	n	-	-	-	y	812	n	-
P. stewartii	n	-	-	y	n	6313	Grouped in another on plasmid	y	812	n	-
S. enterica arizonae	y	y	21122	y	y	6307	Unique	y	812	n	-

S. bongori	y	y	21122	y	y	6307	Unique	y	812	n	-
S. enterica diarizonae	y	y	21122	y	y	6307	Unique	y	812	n	-
S. enterica ser. Enteriditis	y	y	21122	y	y	6307	Unique	y	812	y	25099
S. enterica ser. Paratyphi	y	y	21122	y	y	6307	Unique	y	812	y	25099
S. enterica ser. Senftenberg	y	y	21122	missing *sitC*	y	6307	Unique	y	812	y	25099
S. enterica ser. Typhi	y	y	21122	y	y	6307	Unique	y	812	y	25099
S. enterica ser. Typhimurium	y	y	21122	y	y	6307	Unique	y	812	y	25099
S. glossinidius	n	-	-	n	-	-	-	y	812	n	-
S. praecaptivus	n	-	-	y	n	6306	Grouped in another in genome	y	812	n	-
Y. enterocolitica	n	-	-	y	n	6347	Grouped in another in genome	y	812	n	-
Y. pestis	n	-	-	y	n	7501	Grouped in another in genome	y	812	n	-

[1]Island IDs are specific to our xenoGI analysis outpu

microorganisms

Supplementary Table S2. HilA homolog accession list from representative species

Species	Genome accession	HilA homolog accession
Salmonella Typhi	NC_003198.1	CAD05983.1
Salmonella bongori	NC_015761.1	CCC31553.1
Salmonella Typhimurium LT2	NC_003197.1	AAL21756.1
Chromobacterium violaceum ATCC 12472	NC_005085.1	WP_011136187.1
Chromobacterium vaccinii strain XC0014	NZ_CP022344.1	WP_104947245.1
Chromobacterium sp. IIBBL 274-1	CP029554.1	AXE33394.1
Chromobacterium rhizoryzae strain JP2-74	CP031968.1	AXT48400.1
Escherichia ETT2 EAEC 042	NC_017626.1	WP_001176839.1
Escherichia coli O7:H1 CE10	NC_017646.1	WP_001176825.1
Escherichia coli SMS-3-5	CP000970.1	ACB16655.1
Escherichia fergusonii EFCF056	NZ_CP040805.1	WP_125400638.1
Iodobacter fluviatilis PCH194	NZ_CP025781.1	WP_130105149.1
Escherichia albertii 05-3106	NZ_CP030778.2	WP_113623039.1
Escherichia albertii 2010C-3449	NZ_CP034212.1	WP_151401622.1
Sodalis praecaptivus HS1	NZ_CP006569.1	WP_025420813.1
Plesiomonas shigelloides G5270	NZ_WEKF01000006.1	WP_152108672.1
Plesiomonas shigelloides SPI-1 island	MK256934.1	QBJ27749.1
Burkholderia ubonensis MSMB1157	NZ_LNJU01000002.1	WP_060183501.1
Burkholderia ubonensis MSMB1183	NZ_LPEA01000163.1	WP_060056825.1
Burkholderia ubonensis MSB2020	NZ_LPCG01000024.1	WP_059786159.1
Burkholderia H12714	NZ_NKFM01000111.1	WP_089487916.1
Aeromonas cavernicola	NZ_PGGC01000040.1	WP_100293059.1
Serratia proteamaculans	NZ_SRIA01000021.1	WP_135318288.1
Providencia alcalifaciens 205/92	NZ_JALD01000080.1	WP_051489889.1
Providencia alcalifaciens wls1935	NZ_WLUA01000010.1	WP_154635911.1
Providencia alcalifaciens PAL-2 F90-2004	JACS01000070.1	ETT04766.1
Providencia wls1938	WLTY01000007.1	MTC22217.1
Pseudogulbenkiania ferooxidans	NZ_AVPH01000294.1	WP_021479055.1
Pseudogulbenkiania ferooxidans	AVPH01000288.1	ERD99719.1
Yersinia enterocolitica	WP_083164271.1	WP_083164271.1

Supplementary Table S3. HilCD homolog accession list from best blastx hits

Species	HilC/D homolog accession	Species	HilC/D homolog accession
S. Typhimurium HilC	NP_461788.1	E. albertii	WP_059279567.1
S. bongori HilC	WP_000243993.1	*E. albertii*	WP_059222165.1
C. freundii	WP_003842073.1	*E. albertii*	WP_059257135.1

C. freundii	WP_003845296.1	*E. albertii*	WP_059260052.1
C. freundii	WP_060854950.1	*E. coli*	EMU75596.1
C. werkmanii	WP_121529575.1	*E. coli*	WP_001685873.1
C. werkmanii	WP_150345454.1	*E. coli*	EMV83912.1
E. hoshinae	AOV95787.1	*E. coli*	WP_097343266.1
E. hoshinae	WP_115314437.1	*E. albertii*	WP_072251280.1
C. freundii	WP_114681257.1	*E. coli*	WP_074157410.1
E. coli	WP_001021810.1	*E. coli*	WP_106668560.1
E. coli	WP_112837021.1	*E. coli*	WP_001734729.1
E. coli	WP_077895800.1	*E. coli*	WP_089560272.1
E. coli	EAB0669272.1	*E. coli*	WP_137456390.1
E. coli	WP_064757389.1	*E. coli*	WP_158179605.1
E. coli	WP_097742501.1	*E. coli*	CEJ09698.1
E. coli	WP_096860707.1	*E. coli*	OKT61493.1
Escherichia	WP_131162275.1	*E. coli*	WP_114142805.1
E. coli	WP_157918601.1	*E. coli*	WP_074558869.1
E. coli	WP_089516252.1	*E. coli*	WP_096307300.1
E. coli	WP_089545489.1	*E. coli*	SQK49161.1
E. coli	WP_112076316.1	*E. coli*	EYE30976.1
E. coli	WP_157918601.1	*E. coli*	WP_077698060.1
H. alvei	WP_004091164.1	*E. coli*	WP_115202920.1
Enterobacteriaceae	WP_118938262.1	*E. coli*	WP_137649357.1
S. Typhimurium HilD	NP_461796.1	*E. coli*	WP_001587483.1
S. bongori HilD	WP_000432692.1	*E. coli*	WP_097493316.1
E. tarda	WP_141112179.1	*E. coli*	WP_087895048.1
E. lignolyticus	ADO47274.1	*E. coli*	WP_053263993.1
E. lignolyticus	WP_157865575.1	*E. coli*	WP_089618576.1
E. coli	EMW29479.1	*E. coli*	WP_148444262.1
E. coli	WP_001595107.1	*E. coli*	WP_047647086.1
E. coli	WP_087905903.1	*E. coli*	WP_001380500.1
E. coli	WP_089644598.1	*E. coli*	WP_004097085.1
E. coli	WP_097430211.1	*E. coli*	WP_001695115.1
E. coli	ENA19068.1	*E. coli*	WP_126125596.1
E. coli	WP_001426713.1	*E. coli*	WP_126687956.1
E. coli	WP_089633437.1	*E. coli*	WP_001415033.1
E. coli	WP_137469516.1	*E. coli*	WP_000017442.1
E. albertii	WP_059217848.1	*E. coli*	WP_001717369.1
E. albertii	WP_059225656.1	*E. coli*	WP_071608303.1
E. coli	WP_001743887.1	*E. coli*	KPQ45501.1
E. coli	WP_126716309.1		
